# The psychophysiological mechanisms of real-world time experience

**DOI:** 10.1038/s41598-022-16198-z

**Published:** 2022-07-28

**Authors:** Ruth S. Ogden, Chelsea Dobbins, Kate Slade, Jason McIntyre, Stephen Fairclough

**Affiliations:** 1 School of Psychology, Liverpool John Moores University, Liverpool, L33AF UK; 2 School of Information Technology and Electrical Engineering, University of Queensland, St Lucia, QLD 4072 Australia; 3 Department of Psychology, Lancaster University, Lancaster, LA14YW UK

**Keywords:** Psychology, Human behaviour

## Abstract

Our sense of time is fallible, often resulting in the sensation of time flying by quickly or dragging slowly. It has been suggested that changes in sympathetic (SNS) and parasympathetic nervous system (PNS) activity may influence the perceived passage of time, however this proposition has never been tested during real-world temporal experience. The current study directly tested the relationship between the passage of time and SNS–PNS activity in the real-world. Sixty-seven participants completed a normal day’s activities whilst wearing sensors to capture electrocardiography (ECG), electrodermal activity (EDA) and movement. They also provided hourly rating of the subjective speed at which time was passing. Results revealed that greater SNS activity (e.g., increased heart rate, frequency of phasic skin conductance response) was associated with time passing more quickly. PNS activity was not related to time experience. Whilst the findings support previous suggestions that changes in physiological arousal are associated with distortions to the passage of time, the effects are small and other factors are likely to contribute to real-world temporal experience.

## Introduction

Our sense of time guides our thoughts and actions and influences how we interpret the world around us. An accurate sense of time during day-to-day life is therefore fundamental to our experiences and survival^1^. Despite this, our sense of time is fallible and distortions to the speed at which time feels like it is passing are common^2,3^. Unlike clock measured time, which passes at a constant linear rate, our subjective experience of the speed of time can be influenced by activities and emotional experiences^4^. Therefore, the passage of time is prone to psychological distortion, sometimes passing very quickly or dragging slowly along.

Distortions to the passage of time have significant consequences for health and the economy. The feeling that one does not have enough time, for example, is associated with lower life satisfaction, poorer health outcomes, and risky decision-making^5^. In health and commercial settings, the subjective lengthening of waiting times leads to reduced engagement with healthcare services and poorer customer satisfaction^6^. Finally, in clinical populations and the elderly, distortions to time are associated with distress, symptom exacerbation and impaired social interaction^7,8^. Despite these deleterious effects, little is known about the underlying causes of distortions to the passage of time during everyday life.

One reason that the mechanisms for temporal distortions during real-world activity are poorly understood is that studies of temporal experience during daily life are relatively rare and have generally relied upon self-reported measures of emotion and arousal, rather than objective measures of underlying mechanism^9,10^. For example, in a series of experience sampling method studies, Droit-Volet and colleagues explored the relationship between the passage of time, emotion, arousal and attention to establish factors that predicted temporal experience. Using mobile devices, participants were promoted at regular points in the day to use rating scales to measure the subjective speed of time (i.e., faster than normal–slower than normal) and subjective emotion (positive–negative)^2,3,11–14^. These studies have *generally* demonstrated that self-reported positive emotion is associated with time passing more quickly, whereas self-reported negative emotion is related to a perception that time is passing more slowly^11–13^. Using a similar methodology, Droit-Volet et al.^13^ have also observed associations between self-reported arousal and distortions to the passage of time with greater self-reported arousal being associated with a faster passage of time. During the COVID-19 pandemic, online questionnaires were used to establish how the emotional and social changes induced through the pandemic altered the passage of time^2,3,15,16^. These studies typically used single Likert scales or visual analogue scales to measure the passage of time, and then related these judgments to measures of mood, anxiety depression and stress, activity and lifestyle factors^2,3,15,16^. These studies have generally demonstrated that boredom, social isolation, and stress are associated with a slowing of the passage of time, whereas greater social satisfaction and low stress are associated with time passing more quickly^2,3,15,16^. On the basis of these findings, it is often assumed that changes in physiological arousal, which often accompany fluctuations of self-reported emotions, play an important role in time distortion, but to date this inference has not been tested during real-world activity, using objective measures of physiological arousal.

One way to directly test the relationship between physiological arousal and temporal experience is to objectively measure the activity of the sympathetic nervous system (SNS) and parasympathetic nervous system (PNS) and then explore how changes in SNS and PNS activity are related to distortions to time. This approach has been used in a series of laboratory studies exploring distortions to the perceived duration of very short events (< 1 s). Here, distortions to the perceived duration of highly arousing negative stimuli were predicted by sympathetic nervous system (SNS) reactivity, i.e., greater SNS reactivity being associated with greater distortion to time^17,18^. Conversely, administration of clonidine, which reduces sympathetic tone^19^, and controlled breathing, which increases parasympathetic nervous system (PNS) activity^20^, have been demonstrated to slow the passage of time. These effects can be interpreted within Craig’s^21,22^ homeostatic model of time, which proposes that temporal distortions are associated with concurrent activation of the anterior insular cortex (AIC). The AIC is bilaterally activated during homeostatic regulation^23,24^ and is also active during temporal perception^25^. According to this framework, time is perceived to accelerate during activation of the SNS, which is associated with activation of the right AIC, which distorts time by increasing in the number of temporal units processed. Conversely, Craig speculated that increased activation of the PNS is associated with activation of the mid-insula and left side of the AIC, which may counteract the influence of SNS on time perception, resulting in a slowing of time.

Although Craig’s model and the findings of laboratory studies indicate that some forms of temporal judgement are affected by objective measures of physiological arousal, it is unclear whether these relationships extend to passage of time judgements. This is because short prospective estimates of duration, such as those used in laboratory studies, do not appear to correlate with passage of time judgements and may therefore be based on distinct cognitive and neural processes^11,13^. Furthermore, inconsistent relationships between self-reported arousal/emotion and the passage of time, imply that the relationship may be more complex than a simple mapping of SNS and PNS activation on speeded up or slowed passage of time. For example, observations of accelerations of the passage of time during high levels of engrossment^26^ or flow^27^, in which both SNS and PNS activation can occur^28^, suggest direct mapping may be unlikely. Similarly, contrasting associations between self-reported high arousal and a faster passage of time^11–13^, and increased stress and a slowing of time^2,3^, suggest that increased self-reported arousal may speed up and slowdown the passage of time in different circumstances. Real-world research, in which measures of SNS and PNS activity are related to the experience of time during everyday life, is therefore required to establish whether autonomic activation has any significant association with time perception outside of the laboratory.

The current study sought to establish whether changes in physiological arousal (SNS and PNS activity) were associated with distortions to time during everyday life. The study used a modified version of the experience sampling design developed by Droit-Volet and Wearden^11,12^ in which participants go about their normal daily business whilst periodically reporting their experience of time. Subjective self-report data were collected over a period of 6 h, from early morning to late afternoon. To measure the passage of time, participants rated their subjective experience of the speed of time over the last hour in comparison to normal (very slowly–very quickly). This method of establishing the subjective passage of time is consistent with that used in studies exploring the passage of time during the COVID-19 pandemic^2,3,15,16^, during waiting^29,30^, drug use^31^, and those during normal daily life^32^. Participants also rated the valence of their mood (positive–negative), and their level of engagement (very engaged–very disengaged). Quantitative data were collected from ambulatory psychophysiology sensors using two wearable Shimmer3 units worn on the chest and hand throughout the 6-h period of study. These sensors included a 5-lead electrocardiogram (ECG) unit, which used disposable electrodes worn on the chest and a wrist-worn sensor that recorded electrodermal activity (EDA) from the fingers of the non-dominant hand. Activity levels were recorded from a triaxial accelerometer embedded within the Shimmer3 ECG unit.

Multi-level modelling, conducted in STATA^33^, was utilised to establish the relationship between autonomic activation, movement, and subjective measures of valence and temporal perception. It was hypothesised that physiological arousal would be associated with distortions to the passage of time. Specifically, it was anticipated that increased SNS activation (i.e., higher skin conductance level and a greater number of electrodermal responses) would be associated with a faster passage of time, whereas increases in PNS activity (i.e., increased heart rate variability) would be associated with a slower passage of time.

## Results

**Figure 1:**
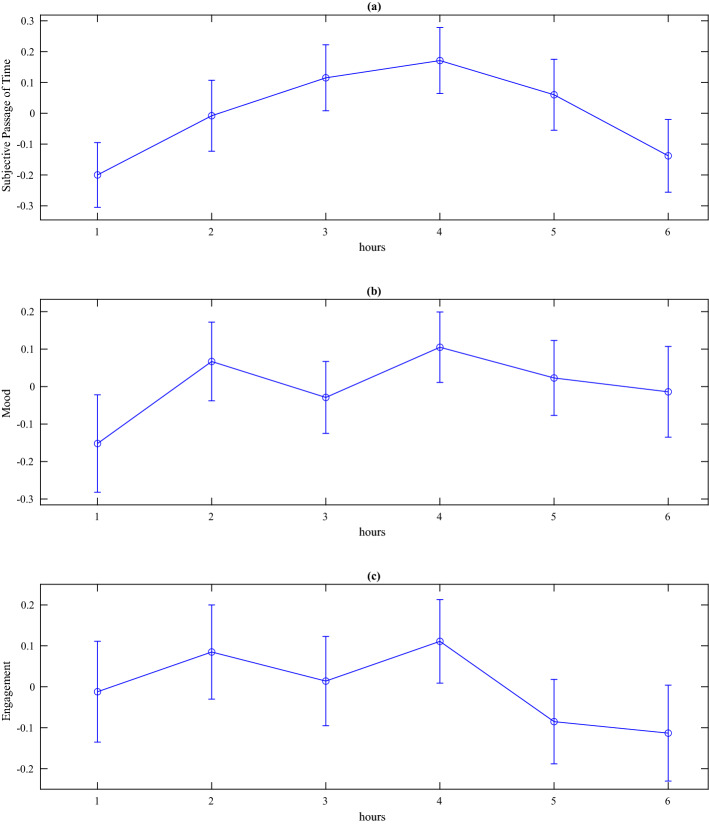
Line plots with error bars for standardised subjective data, (**a**) passage of time judgements (**b**) mood, and (**c**) engagement. Higher scores = time passing quickly (**a**), positive mood (**b**), and very engaged (**c**).

**Figure 2:**
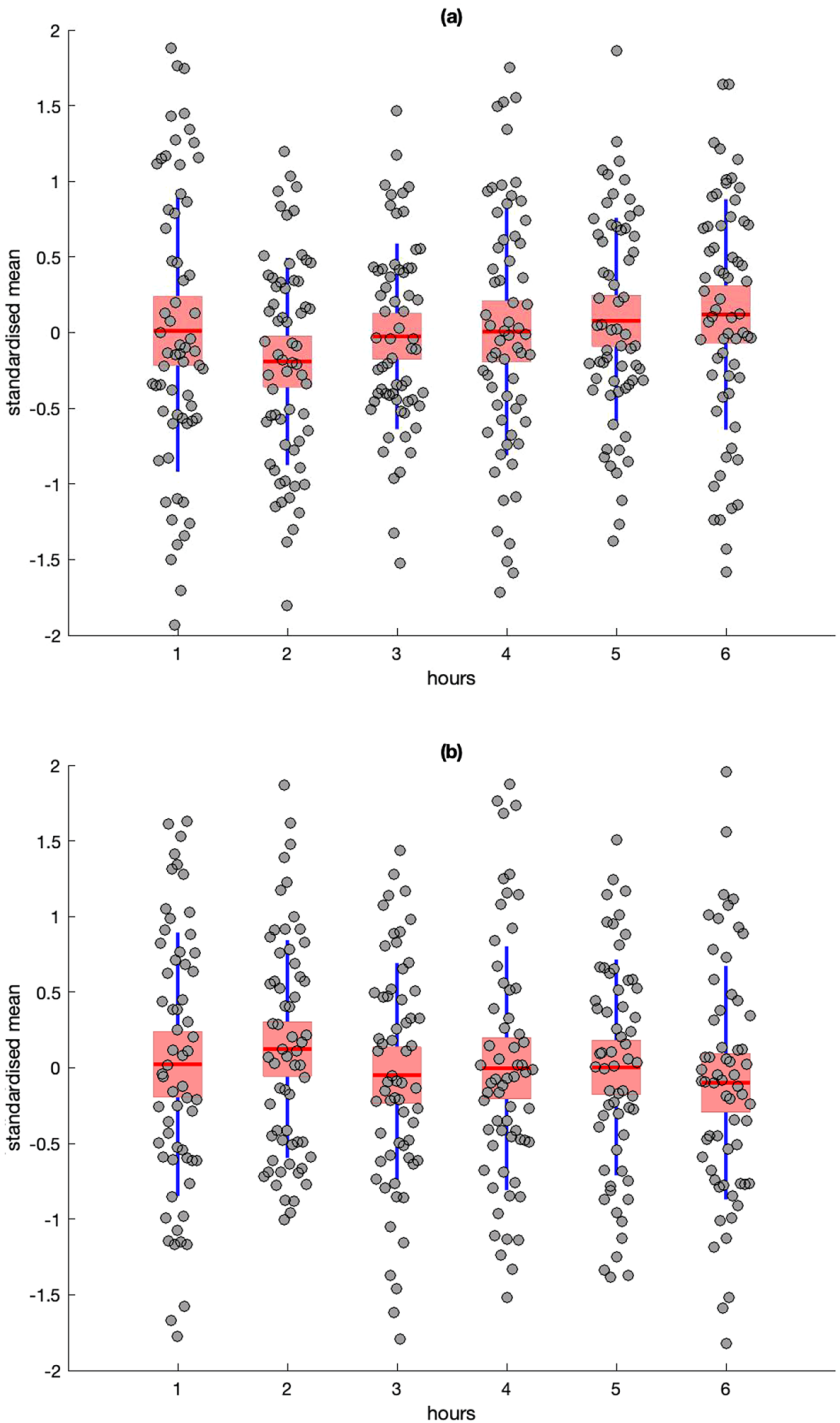
Descriptive statistics for standardised residuals for Heart Rate (HR) (**a**) and RMSSD (**b**). Red line = mean, pink box = standard deviation, blue lines = 95% confidence intervals, grey circles = raw data.

**Figure 3:**
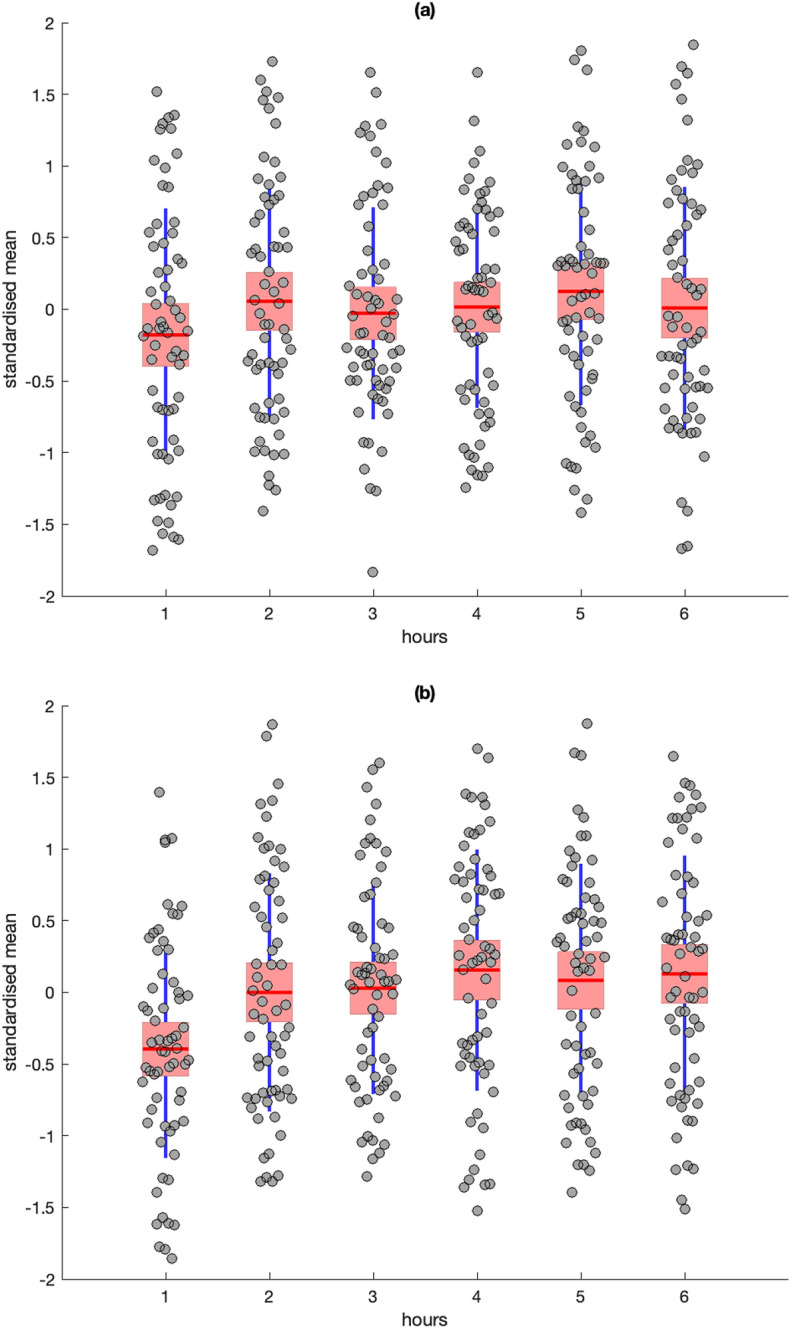
Descriptive statistics for standardised residuals from Skin Conductance Level (SCL) (**a**) and frequency of Skin Conductance Response (SCR) (**b**). Red line = mean, pink box = standard deviation, blue lines = 95% confidence intervals, grey circles = raw data.

In total, data from six participants were excluded from the psychophysiological data due to a sensor failure to record movement, i.e., unable to correct data for effects of movements (3 participants), and inability to calculate the root mean square of successive differences between normal heartbeats (RMSSD) metric during 1 h of data collection due to signal noise (3 participants). The descriptive statistics for the remaining 61 participants, with respect to both subjective self-report data and all four measures of psychophysiology are presented in Figs. 1, 2 and 3 below.

A repeated-measures ANOVA was conducted to test for differences between 6 h of data collection for all subjective measures. Neither analyses of subjective passage of time [*F*(5,62] = 1.07, *p* = .39], subjective mood [*F*(5,62) = .60, *p* = .70], or subjective engagement [*F*(5,62) = .46, *p* = .81] revealed any significant changes across 6 h of data collection.

A repeated-measures ANOVA revealed no significant differences between mean Heart Rate (HR) [*F*(5,58) = 1.12, *p* = .36] or RMSSD [*F*(5,56) = .58, *p* = .71] across the 6 h of data collection.

There was no significant effect of time on Skin Conductance Level (SCL) [*F*(5,58) = .91, *p* = .48], however, the analyses of Skin Conductance Response (SCR) frequency revealed a significant effect [*F*(5,58) = 3.37, *p* = .01, η^2^ = .23]; post-hoc Bonferroni tests indicated that SCR frequency was significantly lower at hour 1 compared to hours 4–6 (*p* < .05) (Fig. 3b).

### Multilevel analysis of predictors of the passage of time

Multilevel model analysis was conducted in STATA version 13^33^ using the XTMIXED command. Variation between participants was considered using a random intercept model, with the random part of the model considering timepoints (level 1) nested within participants (level 2). The fixed part of the model included level 6 explanatory variables of standardized RMSSD, SCL, SCR, HR, mood, and engagement (see Table 1). The random intercept model was chosen because it represented the simplest model that would accurately describe the data. Specifically, participants were likely to vary in their baseline time experience; therefore, including random intercepts allowed this variation to be modelled. Results showed that the overall model was significant, Wald χ^2^ (6) = 12.74, *p* < .05. SCR and HR were significant positive predictors of the passage of time. As shown in Table 1, greater SCR and HR were significantly associated with the subjective experience of time passing more. SCL, RMSSD, mood and engagement were not predictive of the passage of time.

**Table 1:** Outcomes of the multi-level model including standardized and unstandardized betas and Cohen’s f effect size.

	B	β	SE	*p*	95% CI	f
SCR	.14	.10	.07	.047	.01–.27	.15
HR	.15	.10	.07	.04	.01–.29	.16
RMSSD	.08	.08	.07	.24	− .05 to .22	.10
SCL	− .10	− .07	.07	.17	− .23 to .04	.12
Mood	− .10	− .09	.06	.08	− .22 to .01	.14
Engagement	.007	.006	.06	.90	− .11 to .12	.06

## Discussion

The results generally supported the hypothesis that activation of the SNS predicted an increase in the subjective speed at which time passed during everyday life. The multilevel model revealed that increased heart rate and a higher number of SCRs positively predicted the subjective passage of time. The findings therefore demonstrate, for the first time, that real-world temporal experience is modulated, albeit to a small extent, by SNS activity. The current study did not find evidence that PNS activity, self-reported emotion or self-reported engagement were related to distortions to the subjective passage of time.

The findings partially support previous suggestions that physiological arousal can influence passage of time judgments during real-world activity. The effect of SNS activity on temporal experience is not therefore limited to laboratory studies of the processing of short durations^17,18^, but extends to passage of time judgments made during real world activity. The association between increased SNS activity and distortions to time therefore appears to be universal to our temporal experience, affecting the judgment of short and long durations, in the laboratory and the real world. This is regardless of whether judgements are made prospectively or as passage of time judgements, or whether they are made numerically (e.g., estimates in seconds) or as comparative subjective ratings (fast-slow). Whilst this convergence perhaps suggests some commonality in the psychophysiological mechanisms underpinning different forms of temporal judgment, it does not preclude previous suggestions that different forms of temporal judgement evoke different cognitive and neural systems^11,12,34,35^. For example, SNS activity may distort prospective temporal judgements via changes in the raw neural processing of time (e.g. internal clock speed) but alter passage of time judgments via arousal induced changes in attention to time.

Whilst the current study demonstrates that SNS activity is predictive of real-world distortions to the passage of time, SNS activity only accounted for a small proportion of the variance in temporal experience. This suggests that changes in physiological arousal are not the primary cause of real-world distortions to time and that that other factors may be determinant of our real-world temporal experience. Studies of experiences of the passage of time during waiting suggest that variations in temporal awareness may be a critical determinant of variations in distortions to time^29,30^. Here, heightened temporal awareness during waiting is associated with a slowing of the passage of time. Conversely, studies of the passage of time during recreational drug use suggest that an absence of temporal awareness is associated with a speeding up of the passage of time^4^. It is therefore possible that, during the complexity of real-world activity, temporal awareness is a primary determinant of the passage of time. Future research should therefore seek to develop objective measures of temporal awareness to establish their role in subjective experiences of the passage of time.

A further possibility is that the effects of SNS and PNS activity on passage of time judgements was attenuated in the current study by the decision to control for the influence of physical activity on SNS and PNS activity. The influence of physical activity on SNS–PNS activity was controlled for because physical activity itself does not consistently influence passage of time judgements^2,3^ and because of concern that changes in SNS–PNS activity due to physical activity may mask changes in SNS–PNS activity due to psychological processes. Future research should however seek to explore how physiological change due to physical activity may contribute to distortions to the passage of time.

Further attenuation of the effects of SNS and PNS activity could have occurred because of the limitations of real-world ambulatory recording. For example, it is possible that known diurnal influences on heart rate variability could have masked the effect of PNS activity on passage of time judgments^36^, however we did not observe the expected decline of RMSSD from morning to afternoon in our data (Fig. 2b). The same explanation could partly account for the absence of any significant influence from SCL, although both SCL and SCRs are known to be higher in the morning than the afternoon^37^; an effect that was replicated for SCRs in the current study (Fig. 3b). With respect to SCL, it should also be noted that the study utilized dry electrodes to capture EDA, which are more practical for everyday use^38^ but rely on the generation of sweat to act as an electrolyte between the skin and sensor^39^. This dependency can reduce the absolute level of SCL, potentially blunting the sensitivity of this measure, but does not influence the frequency of SCRs to the same extent^40^. Alternatively, there is evidence that frequency of SCRs is more sensitive to changes in sympathetic activation than tonic SCL. A recent study^41^ captured EDA from a large sample across a variety of laboratory tasks and everyday activities, they reported that: (1) SCR frequency was strongly associated with fluctuations in stress and cognitive load compared to SCL, and (2) frequency of SCRs exhibited convergent validity with changes in the pre-ejection period (a cardiovascular marker of sympathetic activation) in comparison to SCL.

As this was a real-world study of everyday life, it is also possible that several other factors may have influenced our data. While the influence of physical activity on autonomic activation was mitigated by our regression-based modelling of individual data sets, levels of autonomic activation can be influenced by other variables^42^. Given that we used dry electrodes for EDA, it would have been useful to monitor ambient temperature, which has a positive association with SCRs^43^, especially as participants could move between indoors and outdoors and within different indoor locations. While there was no significant variation in subjective ratings across the 6 h of data collection, it was noted that subjective estimates of time passing and mood all tended to peak at the fourth hour (Fig. 1), which would generally coincide with 13:00 h. This observation begs a question about how food intake and consumption of beverages, such as coffee, may could have exerted a systematic influence on sympathetic activation at that specific time of the day. Furthermore, it is likely that participants may have socialized in face-to-face settings during this period, enhancing positive mood and engagement. It is therefore important for our finding to be replicated, whilst controlling for these potential influences^44^, and during a data collection window that represents the period between mid-afternoon and evening.

## Conclusion

In conclusion, the current study substantially advances our understanding of why the subjective speed of time distorts during normal daily life. By objectively measuring SNS and PNS activity, we were able to directly test the relationship between physiological arousal and the passage of time during real-world activity. Our finding that increased SNS activity was predictive of an acceleration in the speed of time adds to previous evidence from theoretical models and laboratory studies that SNS activity can alter temporal experience. However, the relatively small contribution of SNS activity to passage of time judgements and the absence of an effect of PNS activity suggested that the relationship between the subjective speed of the passage of time and autonomic activity is perhaps more complex than previously envisaged.

## Method

### Participants

Sixty-seven participants (51 females and 16 males; mean age = 24.39, SD = 5.27) with no known history of cardiac pathology were recruited via email through volunteer sampling from Liverpool John Moores University and the general population. Sample size was determined a-priori based on Quintana’s^45^ review of sampled sizes in studies of heart-rate variability, which indicated that a sample size of 60 was sufficient to detect a medium effect size. Participants were given a £10 shopping voucher in exchange for participation. All participants gave informed written consent. The study was approved by Liverpool John Moores University Research Ethics Committee. The study was conducted in accordance with the principles expressed in the Declaration of Helsinki.

### Materials and apparatus

Physiological Equipment: One mobile Shimmer sensor collected ambulatory ECG signals and movement data. The 5-lead ECG unit was fastened to the participant’s chest via an elasticated belt and was configured at a sampling rate of 1024 Hz. The bipolar limb lead electrodes were placed on the participant’s chest, at the left clavicle, right clavicle, left pelvic bone, and right pelvic bone. The unipolar lead electrode was placed in the centre of the chest at the level of the xiphoid. A second Shimmer3™ sensor collected ambulatory EDA signals, which was secured to the participant’s non-dominant hand, via an elasticated wrist strap, and was configured to sample data at 100.21 Hz. The electrodermal sensors were dry and secured to the proximal phalanx of the participant’s index and middle fingers using Velcro straps. The raw data from 6 h of recording were stored on the internal micro-SD card of each device.

### Procedure

Participants were informed that the aim of the study was to understand how real-world physiological arousal related to self-reported experiences of the passage of time. Participants were not therefore naïve to the study aims. They were instructed that, following the attachment of the physiological sensors, they should leave the laboratory and continue their normal daily activities, except for immersing their bodies or non-dominant hand in water. During their daily activities they should expect to receive a series of text messages every hour asking them to rate (1) how quickly time felt like it was passing in comparison with normal, (2) the valence of their mood, and (3) their level of engagement with current activities. Participants were advised to respond promptly to the messages and were informed that after approximately 6 h they would be asked to return to the laboratory to return the recording equipment.

Each participant took part individually and attended the laboratory at Liverpool John Moores University twice on the day of their participation. On the first visit, the participant provided informed consent. The study was subsequently explained to participants and their mobile telephone number was recorded. All first visits took place between 9 a.m. and 11 a.m.

The two Shimmer sensing units were then attached to the participant’s torso and hand for the recording of ambulatory psychophysiology and movement data. At this point, the experimental start time (T1) was recorded and participants left the laboratory to continue their daily activities. After 60 min, participants received a text message asking them to provide the following ratings, using various 9-point rating scales:

1. In comparison with a typical hour, how quickly did you feel that the last hour passed?

Where 1 represented very slowly, 9 represented very quickly and 5 represented the mid-point, i.e., time passing normally.

(2) Rate your mood during the last hour:

Where 1 represented very negative and 9 represented very positive.

(3) Rate your level of engagement during the last hour:

Where 1 represented very unengaged and 9 represented very engaged.

The response times to the messages sent at T1 were recorded (R1) and 60 min after this a second text message was sent asking the same three questions. The mean delay between questions being sent and responses being sent was 5.16 min (SD = 7.50 min). The mean time of day at T1 when the first set of ratings were received was 11:09. This process was repeated until participation had lasted for 6 h. Participants were then sent a final message reminding them to return to the laboratory. Upon returning to the laboratory, the Shimmer units were removed, and participants were debriefed. In total, participation in the study took approximately 7 h.

### Data analysis

ECG, EDA and activity data were analysed using MATLAB vR2019a. Firstly, data from each participant were separated into six files that were each segmented based on the hour preceding the time at which the participant responded to the hourly text message.

ECG: The raw ECG data were pre-processed and analysed using MATLAB code developed to process raw ambulatory ECG data^46^. The code first filtered the raw ECG data using a Chebyshev Type I second order high and low pass filter, with a cut off frequency between .5 and 200 Hz and a passband ripple of 1 dB. The data were divided into 30-s epochs, in which the R-peaks within the ECG signal were detected and the inter-beat interval (IBI) was calculated. An algorithm subsequently identified missing R-peaks (if the IBI value was greater than 1.5 of the mean) and false positive peaks (if the IBI value was less than .5 of the mean), which were then subsequently corrected based on an interpolation process, see Dobbins and Fairclough (2019)^46^ for full details. Heart rate variability (HRV) was quantified as an index of parasympathetic activation in the form of the root mean square successive difference (RMSSD) metric; this metric has convergent validity with other indices of HRV and is stable across ambulatory situations^47^. Because calculation of the RMSSD metric can be significantly distorted by missing R-peaks^48^, any epoch containing a single missed R-peak (that had not been corrected by interpolation in the algorithm) was rejected for subsequent analysis, leading to the loss of 18.33% of the ECG data. Mean heart rate (HR) in beats-per-minute and RMSSD were calculated for each hour of data collection on the basis of those epochs that remained, however, if removal of ‘bad’ epochs led to an omission of > 70% of available epochs per hour, it was decided that remaining data was unrepresentative of that hour, which led to the removal of three participants from the sample.

When collecting ambulatory data using psychophysiological sensors, it is known that changes in physical movement can influence these signals, e.g., increasing frequency of SCR and inflating HR^41,49^. Therefore, a linear regression was conducted using changes in activity levels to predict HR and RMSSD. This model was calculated on a participant-by-participant basis to correct for any potential elevation of HR (and reduction of RMSSD) due to variations of participants’ activity levels over the 6-h data collection period; the standardised residuals (i.e., HR and RMSSD corrected for movement) from both models were entered into the analyses.

#### EDA

Each of the 6 h-long data files containing a raw EDA signal was: (1) filtered using a Chebyshev second order low pass filter at .5 Hz, and a passband ripple of 1 dB, (2) detrended, and (3) normalised. Ambulatory EDA can be analysed using a variety of techniques^38,50^; for the current study, we wished to distinguish between tonic (skin conductance level: SCL) and phasic (skin conductance responses: SCR) aspects of the EDA signal. We selected a method developed by Greco et al.^51^ that uses convex optimisation to decompose the EDA signal into tonic fluctuations of SCL and frequency of phasic SCRs. The detrended and normalised data were divided into epochs of 10 s for each hour of data collection. The range of normalised data were inspected and any epoch that varied by ± 3.5 standard deviations was rejected as a ‘bad’ epoch; this process removed 5.2% of available epochs for analyses. The remaining epochs were subjected to the convex optimisation process described by Greco et al.^51^ using MATLAB code provided by the authors. Mean SCL and the frequency of SCR were calculated for each 10 s epoch and averaged across each hour to yield an average for SCL and a total frequency count for SCR. Any influence of movement on EDA variables was corrected by conducting a linear regression using changes in movement to predict SCL and SCR, as was performed for the ECG data. This model was calculated on a participant-by-participant basis and the standardised residuals were entered into the analyses for both SCL and SCR.

#### Activity

The Shimmer3 sensor used to capture ECG and worn on the chest contained a triaxial accelerometer, which was used to gather data related to movement and activity. Accelerometer data were sampled at 1024 Hz and used to calculate vector magnitude, i.e., total magnitude of movement across x, y, and z axes. Vector magnitude was calculated for each 30 s epoch and averaged to yield a mean value for each hour of data collection. Technical problems led to the loss of activity data from three participants and their omission from the final data set.

## Supplementary Information

Supplementary Information.

## Data Availability

The data for this study are publicly accessible in the supplementary materials. The code for this study and raw data are not publicly accessible but are available on request by emailing Ruth Ogden r.s.ogden@ljmu.ac.uk. The materials used in these studies are widely available. There is not a preregistration for this study.
